# Poly[[μ-(3-amino­pyridine)-κ^2^ *N*:*N*′-μ-chlorido-chlorido(*N*,*N*′-dimethyl­formamide-κ*O*)nickel(II)] *N*,*N*′-dimethyl­formamide monosolvate]

**DOI:** 10.1107/S1600536812036215

**Published:** 2012-08-25

**Authors:** Chun-Wei Yeh, Chi-Hsiung Jou, Chi-Hui Tsou, Maw-Cherng Suen

**Affiliations:** a Department of Chemistry, Chung-Yuan Christian University, Jhongli 32023, Taiwan; b Department of Materials and Textiles, Oriental Institute of Technology, New Taipei City, Taiwan; c Department of Materials Science and Engineering, National Taiwan University of Science and Technology, Taipei 10607, Taiwan; d Department of Applied Cosmetology, Taoyuan Innovation Institute of Technology, Jhongli 32091, Taiwan

## Abstract

The title compound, {[NiCl_2_(C_5_H_6_N_2_)(C_3_H_7_NO)]·C_3_H_7_NO}_*n*_, is a two-dimensional polymer in which the Ni^II^ atom is coordinated by two N atoms from two 3-amino­pyridine ligands, one O atom from a dimethyl­formamide (DMF) group, one terminal Cl and two bridging Cl atoms in a distorted octa­hedral geometry. The Ni^II^ atoms are bridged by the 3-amino­pyridine ligands [Ni⋯N = 6.7048 (3) Å] and Cl^−^ atoms [Ni⋯N = 3.5698 (3) Å], forming (4,4) two-dimensional nets. The DMF solvent mol­ecule and the non-bridged Cl^−^ ions participate in N—H⋯O and N—H⋯Cl hydrogen bonds with the amino groups.

## Related literature

For background to coordination polymers, see: Kitagawa *et al.* (2004 ▶); Chiang *et al.* (2008 ▶); Yeh *et al.* (2008 ▶, 2009 ▶); Hsu *et al.* (2009 ▶). For related 3-amino­pyridine complexes, see: Zhu & Kitagawa (2002 ▶); Lah & Leban (2005 ▶, 2006 ▶); Kochel (2006 ▶); Wu *et al.* (2005 ▶); Qian & Huang (2006 ▶). 
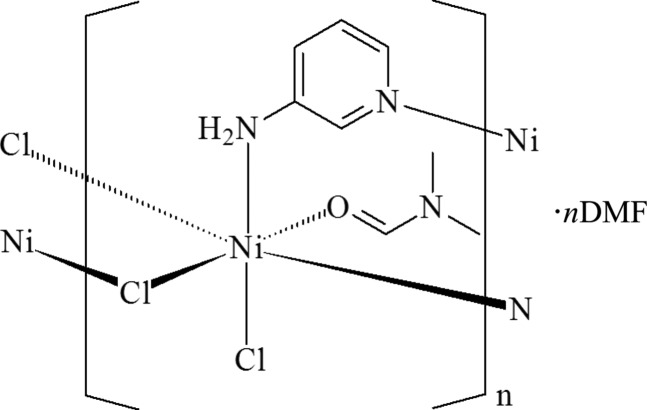


## Experimental

### 

#### Crystal data

[NiCl_2_(C_5_H_6_N_2_)(C_3_H_7_NO)]·C_3_H_7_NO
*M* *_r_* = 369.92
Monoclinic, 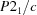

*a* = 10.3684 (2) Å
*b* = 15.0571 (3) Å
*c* = 10.0976 (2) Å
β = 103.832 (2)°
*V* = 1530.70 (5) Å^3^
*Z* = 4
Mo *K*α radiation
μ = 1.62 mm^−1^
*T* = 293 K
0.38 × 0.30 × 0.18 mm

#### Data collection

Bruker SMART APEXII diffractometer
Absorption correction: multi-scan (*SADABS*; Bruker, 2000 ▶) *T*_min_ = 0.628, *T*_max_ = 1.000
6140 measured reflections
2748 independent reflections
2333 reflections with *I* > 2σ(*I*)
*R*_int_ = 0.021

#### Refinement

*R*[*F* ^2^ > 2σ(*F* ^2^)] = 0.025
*wR*(*F* ^2^) = 0.058
*S* = 1.04
2748 reflections
193 parameters
H atoms treated by a mixture of independent and constrained refinement
Δρ_max_ = 0.70 e Å^−3^
Δρ_min_ = −0.34 e Å^−3^

### 

Data collection: *APEX2* (Bruker, 2010 ▶); cell refinement: *SAINT* (Bruker, 2010 ▶); data reduction: *SAINT*; program(s) used to solve structure: *SHELXS97* (Sheldrick, 2008 ▶); program(s) used to refine structure: *SHELXL97* (Sheldrick, 2008 ▶); molecular graphics: *DIAMOND* (Brandenburg, 2010 ▶); software used to prepare material for publication: *SHELXL97*.

## Supplementary Material

Crystal structure: contains datablock(s) I, global. DOI: 10.1107/S1600536812036215/xu5609sup1.cif

Structure factors: contains datablock(s) I. DOI: 10.1107/S1600536812036215/xu5609Isup2.hkl

Additional supplementary materials:  crystallographic information; 3D view; checkCIF report

## Figures and Tables

**Table 1:** Hydrogen-bond geometry (Å, °)

*D*—H⋯*A*	*D*—H	H⋯*A*	*D*⋯*A*	*D*—H⋯*A*
N2—H2*NA*⋯Cl2^i^	0.89 (2)	2.60 (2)	3.4869 (16)	176.4 (1)
N2—H2*NB*⋯O2^ii^	0.84 (2)	2.11 (2)	2.925 (2)	173.3 (1)

Symmetry codes: (i) 
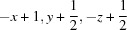
; (ii) 
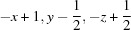
.

**Fig. 1.**
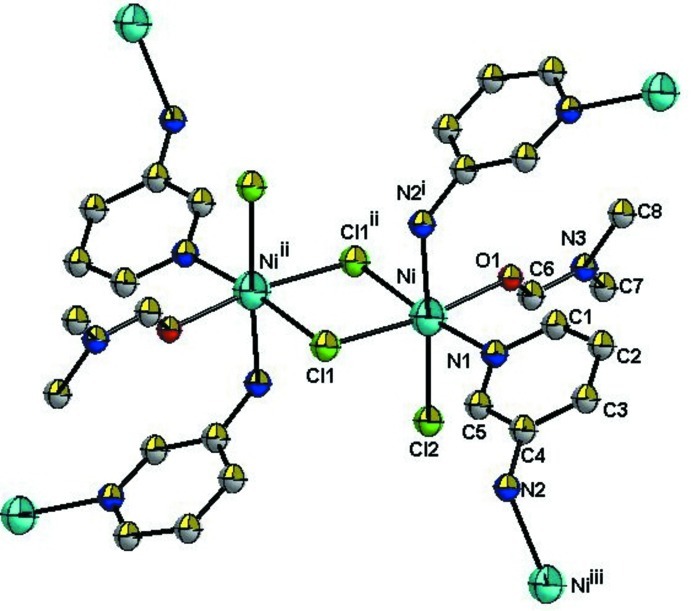
A portion of the two-dimensional grid. Ellipsoids are drawn at 30% probability level. [Symmetry codes: (i) x,-y + 3/2,z + 1/2;(ii) -x + 1,-y + 1,-z + 1; (iii) x,-y + 3/2,z - 1/2.]

**Fig. 2.**
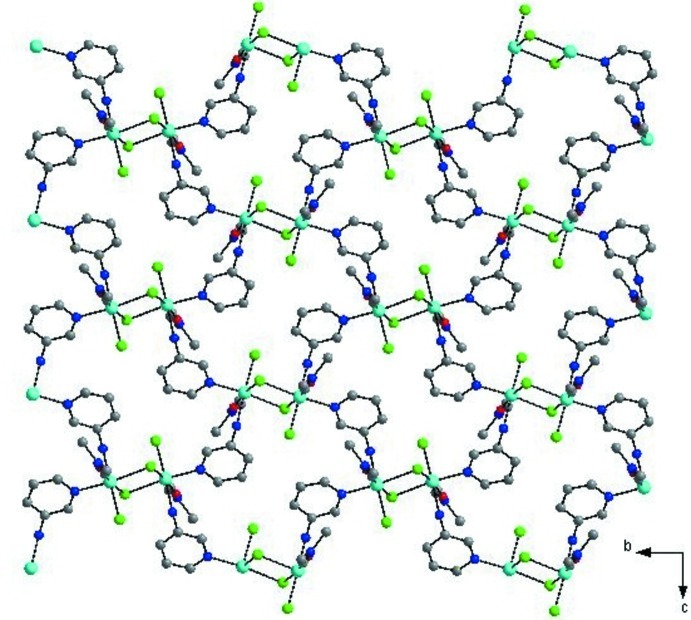
The view shows the two-dimensional pleated (4,4) net.

## supplementary crystallographic information

### Comment

The synthesis of metal coordination polymers has been a subject of intense research due to their interesting structural chemistry and potential applications in gas storage, separation, catalysis, magnetism, luminescence, and drug delivery (Kitagawa *et al.*, 2004). Roles of anion, solvent and ligand comformations in self-assembly of coordination complexes containing polydentate nitrogen ligands are very intersting (Chiang *et al.*, 2008; Yeh *et al.*, 2008; Hsu *et al.*, 2009; Yeh *et al.*, 2009). In the past, the 3-aminopyridine ligands have been subjected to many studies of its coordination ability to structure chemistry (Zhu *et al.*, 2002; Lah & Leban, 2005; Lah & Leban, 2006; Kochel, 2006; Wu *et al.*, 2005; Qian *et al.*, 2006). The Ni^2+^ cations are coordinated with two N atoms from two 3-aminopyridine (*L*) ligands, one O atom from dimethylformamide group, one terminal Cl and two bridging Cl atoms (Fig. 1). The Ni···Ni distances separated by the bridging Cl*^-^* anions and *L* groups are 3.5698 (3) and 6.7048 (3) Å, while the unit of dinuclear [Ni_2_Cl_2_] cores are forming (4,4) polymeric nets (Fig. 2). The co-crystallized DMF molecules and terminal Cl^-^ ions are interacted with the amino hydrogen atoms forming N—H···O and N—H···Cl inter– and intra–molecular hydrogen bonds (Tab. 1).

### Experimental

An aquous solution (5.0 ml) of nickel chloride (1.0 mmol) was layered carefully over a mixed CH_3_OH/DMF solution (5.0 ml, 1:1) of 3-aminopyridine (1.0 mmol) in a tube. Green crystals were obtained after several weeks. These were washed with methanol and collected in 72.8% yield.

### Refinement

H atoms bound to C atoms were placed in idealized positions and constrained to ride on their parent atoms, with C—H = 0.93 - 0.96 A, and with *U*_iso_(H) = 1.2 or 1.5 *U*_eq_(C). The amine hydrogen atoms (H2NA and H2NB) was freely refined.

### Crystal data

**Table:**

[NiCl_2_(C_5_H_6_N_2_)(C_3_H_7_NO)]·C_3_H_7_NO	*F*(000) = 768
*M**_r_* = 369.92	*D*_x_ = 1.605 Mg m^−^^3^
Monoclinic, *P*2_1_/*c*	Mo *K*α radiation, λ = 0.71073 Å
Hall symbol: -P 2ybc	Cell parameters from 4748 reflections
*a* = 10.3684 (2) Å	θ = 3.2–29.0°
*b* = 15.0571 (3) Å	µ = 1.62 mm^−^^1^
*c* = 10.0976 (2) Å	*T* = 293 K
β = 103.832 (2)°	Plate, green
*V* = 1530.70 (5) Å^3^	0.38 × 0.30 × 0.18 mm
*Z* = 4	

### Data collection

**Table:**

Bruker SMART APEXII diffractometer	2748 independent reflections
Radiation source: fine-focus sealed tube	2333 reflections with *I* > 2σ(*I*)
Graphite monochromator	*R*_int_ = 0.021
phi and ω scans	θ_max_ = 25.2°, θ_min_ = 3.4°
Absorption correction: multi-scan (*SADABS*; Bruker, 2000)	*h* = −10→12
*T*_min_ = 0.628, *T*_max_ = 1.000	*k* = −16→18
6140 measured reflections	*l* = −12→11

### Refinement

**Table:**

Refinement on *F*^2^	Primary atom site location: structure-invariant direct methods
Least-squares matrix: full	Secondary atom site location: difference Fourier map
*R*[*F*^2^ > 2σ(*F*^2^)] = 0.025	Hydrogen site location: inferred from neighbouring sites
*wR*(*F*^2^) = 0.058	H atoms treated by a mixture of independent and constrained refinement
*S* = 1.04	*w* = 1/[σ^2^(*F*_o_^2^) + (0.0322*P*)^2^] where *P* = (*F*_o_^2^ + 2*F*_c_^2^)/3
2748 reflections	(Δ/σ)_max_ = 0.001
193 parameters	Δρ_max_ = 0.70 e Å^−^^3^
0 restraints	Δρ_min_ = −0.34 e Å^−^^3^

### Special details

**Table:**

Geometry. All e.s.d.'s (except the e.s.d. in the dihedral angle between two l.s. planes) are estimated using the full covariance matrix. The cell e.s.d.'s are taken into account individually in the estimation of e.s.d.'s in distances, angles and torsion angles; correlations between e.s.d.'s in cell parameters are only used when they are defined by crystal symmetry. An approximate (isotropic) treatment of cell e.s.d.'s is used for estimating e.s.d.'s involving l.s. planes.
Refinement. Refinement of *F*^2^ against ALL reflections. The weighted *R*-factor *wR* and goodness of fit *S* are based on *F*^2^, conventional *R*-factors *R* are based on *F*, with *F* set to zero for negative *F*^2^. The threshold expression of *F*^2^ > σ(*F*^2^) is used only for calculating *R*-factors(gt) *etc*. and is not relevant to the choice of reflections for refinement. *R*-factors based on *F*^2^ are statistically about twice as large as those based on *F*, and *R*- factors based on ALL data will be even larger.

### Fractional atomic coordinates and isotropic or equivalent isotropic displacement parameters (Å^2^)

**Table:**

	*x*	*y*	*z*	*U*_iso_*/*U*_eq_	
Ni	0.41415 (2)	0.603502 (16)	0.48905 (2)	0.00775 (9)	
C1	0.38923 (19)	0.80038 (13)	0.50209 (19)	0.0102 (4)	
H1A	0.3489	0.7849	0.5718	0.012*	
C2	0.4014 (2)	0.88919 (13)	0.4737 (2)	0.0125 (4)	
H2A	0.3717	0.9325	0.5251	0.015*	
C3	0.4579 (2)	0.91299 (13)	0.3683 (2)	0.0120 (4)	
H3A	0.4655	0.9725	0.3466	0.014*	
C4	0.50335 (19)	0.84671 (13)	0.29533 (19)	0.0086 (4)	
C5	0.49098 (19)	0.75896 (13)	0.33264 (19)	0.0089 (4)	
H5A	0.5242	0.7146	0.2860	0.011*	
C6	0.1441 (2)	0.62218 (13)	0.5369 (2)	0.0127 (4)	
H6A	0.1190	0.5851	0.4614	0.015*	
C7	−0.0851 (2)	0.61693 (15)	0.5540 (3)	0.0222 (5)	
H7A	−0.0954	0.5838	0.4709	0.033*	
H7B	−0.1451	0.6665	0.5390	0.033*	
H7C	−0.1045	0.5793	0.6235	0.033*	
C8	0.0852 (2)	0.70808 (15)	0.7160 (2)	0.0185 (5)	
H8A	0.1524	0.7493	0.7047	0.028*	
H8B	0.1179	0.6734	0.7968	0.028*	
H8C	0.0074	0.7401	0.7241	0.028*	
C9	0.2418 (2)	1.14619 (14)	0.3491 (3)	0.0210 (5)	
H9A	0.2665	1.1173	0.2775	0.025*	
C10	0.1142 (2)	1.01507 (16)	0.3680 (3)	0.0342 (6)	
H10A	0.1512	0.9948	0.2949	0.051*	
H10B	0.0196	1.0210	0.3358	0.051*	
H10C	0.1338	0.9730	0.4414	0.051*	
C11	0.1376 (3)	1.13771 (16)	0.5363 (2)	0.0274 (6)	
H11A	0.1539	1.0943	0.6080	0.041*	
H11B	0.0455	1.1541	0.5146	0.041*	
H11C	0.1913	1.1893	0.5657	0.041*	
N1	0.43323 (16)	0.73532 (11)	0.43310 (16)	0.0090 (4)	
N2	0.55335 (18)	0.86856 (12)	0.18049 (17)	0.0093 (4)	
H2NA	0.596 (2)	0.9204 (15)	0.190 (2)	0.010 (6)*	
H2NB	0.600 (2)	0.8292 (16)	0.160 (2)	0.019 (7)*	
N3	0.05143 (17)	0.64924 (11)	0.59758 (17)	0.0140 (4)	
N4	0.17122 (19)	1.10045 (12)	0.41599 (19)	0.0212 (4)	
O1	0.26235 (14)	0.64348 (8)	0.57502 (14)	0.0120 (3)	
O2	0.27851 (16)	1.22267 (10)	0.37064 (17)	0.0267 (4)	
Cl1	0.60647 (5)	0.54783 (3)	0.42042 (5)	0.00949 (12)	
Cl2	0.26678 (5)	0.56642 (3)	0.27259 (5)	0.01240 (12)	

### Atomic displacement parameters (Å^2^)

**Table:**

	*U*^11^	*U*^22^	*U*^33^	*U*^12^	*U*^13^	*U*^23^
Ni	0.00809 (14)	0.00705 (14)	0.00920 (14)	−0.00007 (11)	0.00421 (10)	−0.00004 (10)
C1	0.0088 (10)	0.0124 (11)	0.0097 (10)	0.0003 (9)	0.0028 (8)	−0.0004 (8)
C2	0.0141 (11)	0.0122 (11)	0.0117 (10)	0.0013 (9)	0.0038 (9)	−0.0035 (9)
C3	0.0148 (11)	0.0082 (10)	0.0124 (10)	−0.0016 (9)	0.0021 (9)	0.0005 (8)
C4	0.0061 (10)	0.0118 (10)	0.0076 (9)	−0.0013 (8)	0.0008 (8)	0.0007 (8)
C5	0.0065 (10)	0.0109 (10)	0.0091 (9)	0.0014 (8)	0.0015 (8)	−0.0007 (8)
C6	0.0148 (11)	0.0097 (10)	0.0144 (10)	−0.0005 (9)	0.0052 (9)	0.0022 (9)
C7	0.0140 (12)	0.0201 (12)	0.0347 (13)	−0.0027 (10)	0.0104 (10)	−0.0025 (11)
C8	0.0163 (12)	0.0195 (12)	0.0228 (12)	0.0017 (10)	0.0110 (10)	−0.0030 (10)
C9	0.0066 (10)	0.0182 (13)	0.0414 (14)	−0.0015 (10)	0.0121 (10)	−0.0117 (11)
C10	0.0259 (14)	0.0224 (14)	0.0514 (17)	−0.0045 (11)	0.0036 (13)	−0.0057 (13)
C11	0.0332 (14)	0.0287 (14)	0.0242 (13)	0.0007 (12)	0.0145 (11)	0.0035 (11)
N1	0.0074 (8)	0.0099 (8)	0.0090 (8)	0.0008 (7)	0.0008 (7)	0.0002 (7)
N2	0.0117 (9)	0.0057 (9)	0.0118 (9)	0.0007 (8)	0.0055 (7)	−0.0001 (7)
N3	0.0098 (9)	0.0136 (9)	0.0208 (10)	−0.0002 (8)	0.0079 (8)	−0.0010 (8)
N4	0.0225 (11)	0.0159 (10)	0.0262 (10)	0.0018 (9)	0.0075 (9)	0.0009 (8)
O1	0.0110 (7)	0.0116 (7)	0.0148 (7)	−0.0002 (6)	0.0059 (6)	0.0004 (6)
O2	0.0270 (10)	0.0195 (9)	0.0385 (10)	−0.0038 (8)	0.0174 (8)	0.0034 (8)
Cl1	0.0099 (2)	0.0079 (2)	0.0125 (2)	−0.0002 (2)	0.00632 (19)	0.00006 (19)
Cl2	0.0128 (3)	0.0128 (3)	0.0116 (2)	−0.0007 (2)	0.0029 (2)	−0.0008 (2)

### Geometric parameters (Å, º)

**Table:**

Ni—O1	2.0607 (13)	C7—H7B	0.9600
Ni—N1	2.0860 (16)	C7—H7C	0.9600
Ni—N2^i^	2.1593 (17)	C8—N3	1.462 (3)
Ni—Cl1	2.4124 (5)	C8—H8A	0.9600
Ni—Cl2	2.4139 (5)	C8—H8B	0.9600
Ni—Cl1^ii^	2.4833 (5)	C8—H8C	0.9600
C1—N1	1.343 (2)	C9—O2	1.216 (2)
C1—C2	1.380 (3)	C9—N4	1.305 (3)
C1—H1A	0.9300	C9—H9A	0.9300
C2—C3	1.380 (3)	C10—N4	1.449 (3)
C2—H2A	0.9300	C10—H10A	0.9600
C3—C4	1.388 (3)	C10—H10B	0.9600
C3—H3A	0.9300	C10—H10C	0.9600
C4—C5	1.388 (3)	C11—N4	1.454 (3)
C4—N2	1.417 (2)	C11—H11A	0.9600
C5—N1	1.343 (2)	C11—H11B	0.9600
C5—H5A	0.9300	C11—H11C	0.9600
C6—O1	1.236 (2)	N2—Ni^iii^	2.1593 (17)
C6—N3	1.322 (3)	N2—H2NA	0.89 (2)
C6—H6A	0.9300	N2—H2NB	0.82 (2)
C7—N3	1.462 (3)	Cl1—Ni^ii^	2.4833 (5)
C7—H7A	0.9600		
			
O1—Ni—N1	88.14 (6)	N3—C8—H8A	109.5
O1—Ni—N2^i^	88.87 (6)	N3—C8—H8B	109.5
N1—Ni—N2^i^	88.32 (7)	H8A—C8—H8B	109.5
O1—Ni—Cl1	171.70 (4)	N3—C8—H8C	109.5
N1—Ni—Cl1	96.57 (5)	H8A—C8—H8C	109.5
N2^i^—Ni—Cl1	84.46 (5)	H8B—C8—H8C	109.5
O1—Ni—Cl2	93.85 (4)	O2—C9—N4	126.7 (2)
N1—Ni—Cl2	93.17 (5)	O2—C9—H9A	116.7
N2^i^—Ni—Cl2	176.93 (5)	N4—C9—H9A	116.7
Cl1—Ni—Cl2	92.710 (18)	N4—C10—H10A	109.5
O1—Ni—Cl1^ii^	88.36 (4)	N4—C10—H10B	109.5
N1—Ni—Cl1^ii^	174.19 (4)	H10A—C10—H10B	109.5
N2^i^—Ni—Cl1^ii^	86.97 (5)	N4—C10—H10C	109.5
Cl1—Ni—Cl1^ii^	86.372 (17)	H10A—C10—H10C	109.5
Cl2—Ni—Cl1^ii^	91.690 (18)	H10B—C10—H10C	109.5
N1—C1—C2	122.69 (18)	N4—C11—H11A	109.5
N1—C1—H1A	118.7	N4—C11—H11B	109.5
C2—C1—H1A	118.7	H11A—C11—H11B	109.5
C1—C2—C3	119.24 (19)	N4—C11—H11C	109.5
C1—C2—H2A	120.4	H11A—C11—H11C	109.5
C3—C2—H2A	120.4	H11B—C11—H11C	109.5
C2—C3—C4	118.92 (19)	C5—N1—C1	117.80 (17)
C2—C3—H3A	120.5	C5—N1—Ni	123.02 (13)
C4—C3—H3A	120.5	C1—N1—Ni	119.17 (13)
C3—C4—C5	118.36 (18)	C4—N2—Ni^iii^	118.73 (13)
C3—C4—N2	120.29 (18)	C4—N2—H2NA	112.6 (13)
C5—C4—N2	121.23 (18)	Ni^iii^—N2—H2NA	97.6 (14)
N1—C5—C4	122.94 (18)	C4—N2—H2NB	112.8 (16)
N1—C5—H5A	118.5	Ni^iii^—N2—H2NB	102.9 (16)
C4—C5—H5A	118.5	H2NA—N2—H2NB	111 (2)
O1—C6—N3	123.51 (19)	C6—N3—C7	121.13 (18)
O1—C6—H6A	118.2	C6—N3—C8	120.52 (18)
N3—C6—H6A	118.2	C7—N3—C8	118.24 (17)
N3—C7—H7A	109.5	C9—N4—C10	122.0 (2)
N3—C7—H7B	109.5	C9—N4—C11	120.33 (19)
H7A—C7—H7B	109.5	C10—N4—C11	117.48 (19)
N3—C7—H7C	109.5	C6—O1—Ni	126.39 (13)
H7A—C7—H7C	109.5	Ni—Cl1—Ni^ii^	93.628 (17)
H7B—C7—H7C	109.5		

Symmetry codes: (i) *x*, −*y*+3/2, *z*+1/2; (ii) −*x*+1, −*y*+1, −*z*+1; (iii) *x*, −*y*+3/2, *z*−1/2.

### Hydrogen-bond geometry (Å, º)

**Table:**

*D*—H···*A*	*D*—H	H···*A*	*D*···*A*	*D*—H···*A*
N2—H2*NA*···Cl2^iv^	0.89 (2)	2.60 (2)	3.4869 (16)	176.4 (1)
N2—H2*NB*···O2^v^	0.84 (2)	2.11 (2)	2.925 (2)	173.3 (1)

Symmetry codes: (iv) −*x*+1, *y*+1/2, −*z*+1/2; (v) −*x*+1, *y*−1/2, −*z*+1/2.
